# Effects of the *Bradyrhizobium japonicum* *waaL* (*rfaL*) Gene on Hydrophobicity, Motility, Stress Tolerance, and Symbiotic Relationship with Soybeans

**DOI:** 10.3390/ijms160816778

**Published:** 2015-07-23

**Authors:** Jun-Gu Noh, Han-Eul Jeon, Jae-Seong So, Woo-Suk Chang

**Affiliations:** 1 Department of Biological Engineering, Inha University, Incheon 402-751, Korea; E-Mails: dynaroo@naver.com (J.-G.N.); haneul7760@hanmail.net (H.-E.J.); sjaeseon@inha.ac.kr (J.-S.S.); 2 Department of Biology, University of Texas, Arlington, TX 76019, USA; 3 Division of Biotechnology, College of Environmental and Bioresource Sciences, Chonbuk National University, Iksan 570-752, Korea

**Keywords:** symbiotic nitrogen fixation, soybean symbiont *Bradyrhizobium japonicum*, lipopolysaccharide (LPS), *rfaL*, *waaL*, *O*-antigen ligase, stress responses

## Abstract

We cloned and sequenced the *waaL* (*rfaL*) gene from *Bradyrhizobium japonicum*, which infects soybean and forms nitrogen-fixing nodules on soybean roots. *waaL* has been extensively studied in the lipopolysaccharide (LPS) biosynthesis of enteric bacteria, but little is known about its function in (brady)rhizobial LPS architecture. To characterize its role as *O*-antigen ligase in the LPS biosynthesis pathway, we constructed a *waaL* knock-out mutant and its complemented strain named JS015 and CS015, respectively. LPS analysis showed that an LPS structure of JS015 is deficient in *O*-antigen as compared to that of the wild type and complemented strain CS015, suggesting that WaaL ligates the *O*-antigen to lipid A-core oligosaccharide to form a complete LPS. JS015 also revealed increased cell surface hydrophobicity, but it showed decreased motility in soft agar plates. In addition to the alteration in cell surface properties, disruption of the *waaL* gene caused increased sensitivity of JS015 to hydrogen peroxide, osmotic pressure, and novobiocin. Specifically, plant tests revealed that JS015 failed to nodulate the host plant soybean, indicating that the rhizobial *waaL* gene is responsible for the establishment of a symbiotic relationship between soybean and *B. japonicum*.

## 1. Introduction

The soil bacterium *Bradyrhizobium japonicum* symbiotically infects its host plant soybean (*Glycine max*, L. Merr) and forms nitrogen-fixing nodules on soybean roots. The first step in this nodulation process could be signal exchanges between two partners following bacterial attachment to root hairs of soybeans, which requires intimate bacterial cell-to-host cell interactions [1,2,3]. Exchange of signaling molecules such as plant-derived isoflavonoids and bacterium-derived Nod factors induces multiple changes in genetic and physiological properties of both partners [4,5,6,7,8]. Included are expression of nodulation genes, root hair curling, infection thread formation, and ultimately nodule formation. Specifically, surface components of *B. japonicum* cells can be an important factor that determines infection capability and nodulation feasibility.

Lipopolysaccharide (LPS) is a major component found in rhizobial cell envelope and has been known to be involved in establishing a symbiotic relationship with leguminous plants. Rhizobial LPS structure and genes involved in its biosynthesis have been elucidated: three distinct regions of a lipid A, a core oligosaccharide, and an *O*-antigen side chain polysaccharide [9]. Several mutation studies on LPS biosynthesis pathways revealed that LPS mutants either failed to form normal nitrogen-fixing nodules or formed non-nitrogen-fixing pseudonodules [10,11,12,13]. In addition, our mutant studies on the 5.5 kb LPS-gene region previously identified in *B. japonicum* 61A101C showed that mutation of either *rfaD*, *rfaF*, *lpcC*, or *galE* encoding heptose epimerase, heptosyl transferase, mannosyl transferase, or glucose epimerase, respectively, affects initiation and maintenance of the nodulation process [14,15,16,17,18]. Here, we further analyze the downstream region of *rfaD* and identify another open reading frame (ORF), *waaL* (formerly *rfaL*), presumably encoding *O*-antigen ligase in *B. japonicum*.

In enteric bacteria, WaaL is involved in LPS-core assembly and ligates the *O*-antigen to lipid A-core oligosaccharide to form a complete LPS [19]. Interestingly, WaaL proteins of *Escherichia coli* K-12 and *Salmonella* *enterica* serovar Typhimurium do not share substantial primary-sequence similarity, but they have strikingly similar hydropathy plot patterns [20]. Both proteins appear to be integral membrane proteins comprising 10 or more potential membrane-spanning domains [19,20]. WaaL proteins have also been identified in rhizobia [21] as well as many other bacterial species, including *Vibrio fischeri* [22], *Pseudomonas aeruginosa* [23], and *Erwinia amylovora* [24]. However, little is known about the role of *waaL* in the symbiotic relationship between leguminous plants and nitrogen-fixing rhizobia, especially soybean endosymbiont *B. japonicum*. Here, we characterize the *waaL* gene and thereby extend our list of genes involved in the *B. japonicum* LPS biosynthesis. We constructed a *waaL* mutant strain to examine and compare its cell surface properties and symbiotic capability with the wild type. We conclude that *waaL* is another essential gene responsible for the successful symbiotic nitrogen fixation.

## 2. Results and Discussion

### 2.1. Identification of the B. japonicum waaL (rfaL) Gene

In a previous study, we identified a 5.5-kb gene region involved in LPS synthesis of *B. japonicum* 61A101C and characterized *rfaD*, *rfaF*, *lpcC*, and *galE* encoding heptose epimerase, heptosyl transferase, mannosyl transferase, and glucose epimerase, respectively, [14,15,16,17,18]. Further sequence analysis of the downstream region of *rfaD* revealed an additional ORF whose deduced amino acid sequences (426 amino acids in length) exhibited 100% identity with an *rfaL* gene, encoding an *O*-antigen ligase, from *Bradyrhizobium elkanii*. Interestingly, this BLAST search (http://blast.ncbi.nlm.nih.gov/Blast.cgi) also showed significant similarities with *rfaL* genes from a number of *Bradyrhizobium* species including *B. liaoningense*, *B. yuanmingense*, *B. oligotrophicum*, and *B. japonicum*. Similarities of the top 100 hits range from 64% to 99%. The same species, but different strain *B. japonicum* USDA110 possesses a gene locus with 82% similarity. Surprisingly, this locus (bll5926) was annotated as an unknown protein in Rhizobase (http://genome.microbedb.jp/RhizoBase).

**Figure 1:**
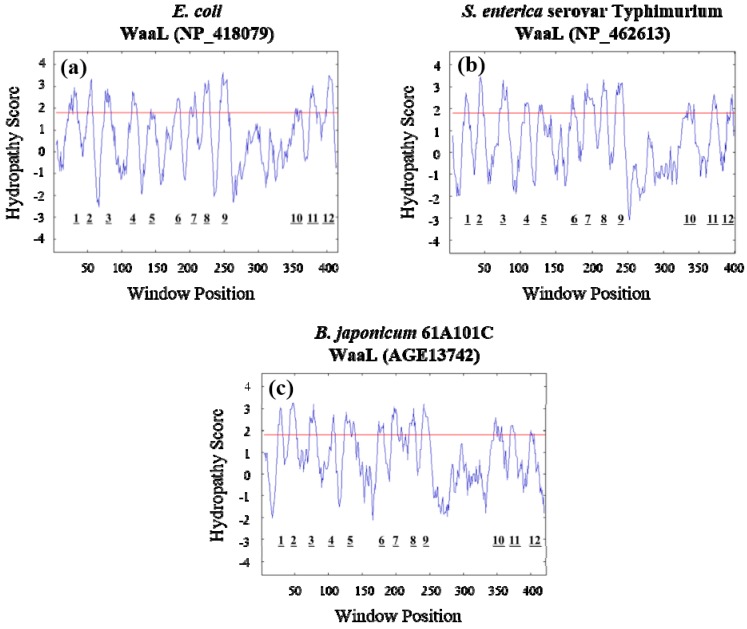
Comparison of hydropathy plots of WaaL proteins from *E. coli* (**a**); *S.* *enterica* serovar Typhimurium (**b**); and *B. japonicum* 61A101C (**c**). The *x-*axis represents the amino acid residue position, while the *y-*axis represents the relative hydrophobicity score. Peaks above the red line indicate potential membrane-spanning domains. Each protein contains 12 potential membrane-spanning domains.

In addition, the BLAST search result demonstrates that most, if not all, of top 100 hits contain Wzy_C superfamily domain (pfam04932) whose sequence is located between 200th and 354th amino acid positions. This domain is known to be involved in the synthesis of *O*-antigen and thus representing *O*-antigen ligases such as enteric WaaL (previously known as RfaL); however, none of the top 100 hits is from enteric bacteria including *E. coli* and *Salmonella*. Therefore, we directly compared amino acid sequences of 61A101C *rfaL* with those of *E. coli* *waaL*. Their sequence homology revealed only *ca.* 14% amino acid identity. This could be a common feature that WaaL proteins are highly variable, even among different serotypes within the same species [25]. Despite the low sequence similarity, the hydropathy profile of the 61A101C RfaL protein was highly similar to WaaL proteins from *E. coli* and *Salmonella* (Figure 1), suggesting that rhizobial RfaL and enteric WaaL proteins have similar enzymatic function. Hydropathy profiles have been used to identify potential genes encoding WaaL proteins [22,26]. Based on the BLAST search and hydropathy profile analysis, we suggest that the *B. japonicum* 61A101C *rfaL* encodes an *O*-antigen ligase and its function is likely in agreement with other enteric WaaL proteins. Moreover, hereafter we rename *rfaL* to *waaL*, at least, in rhizobial strains because the *waa* locus is a new gene name related to LPS biosynthesis.

### 2.2. A B. japonicum waaL Mutant Shows Different LPS Profiles

**Figure 2:**
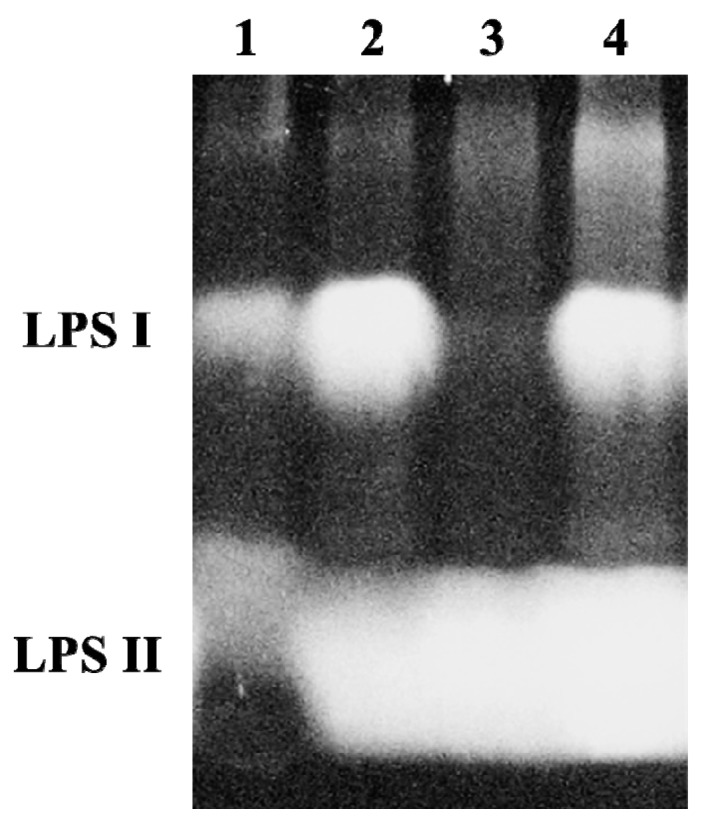
SDS-PAGE of LPS from wild-type, *waaL* knock-out mutant, and complemented strains. LPS I is the high molecular weight form of the LPS, which contains the *O*-antigen. LPS II lacks the *O*-antigen. Lane 1, LPS standard from *E. coli* serotype 055:B5 (Invitrogen, Carlsbad, CA, USA); Lane 2, 61A101C; Lane 3, JS015; Lane 4, CS015.

To characterize a function of *waaL* in the *B. japonicum* LPS biosynthesis, a *waaL* mutant (JS015) and its complemented strain (CS015) were constructed as described in the experimental section and their LPS profiles were compared to the wild type (Figure 2). SDS-PAGE analysis showed that the wild-type LPS contained both LPS-I (*i.e.*, high molecular weight) and LPS-II (*i.e.*, low molecular weight) bands, whereas the JS015 LPS displayed only the LPS-II band, which is an indicative of lacking *O*-antigen. It has been known for *B. japonicum* and *Rhizobium phaseoli* that LPS-I and LPS-II bands represent a complete form of LPS and truncated LPS (*i.e.*, LPS without *O*-antigen), respectively [1,7].

Our finding indicated that the abnormal LPS structure lacking *O*-antigen present in JS015 could be due to the loss of *O*-antigen ligase activity. The LPS profile of CS015 was found to be identical to that of the wild type (Figure 2). Thus, the results confirmed the ligase role of WaaL to connect the *O*-antigen to the outer core of LPS in *B. japonicum*.

### 2.3. Lack of O-Antigen Increases the Cell Surface Hydrophobicity

**Figure 3:**
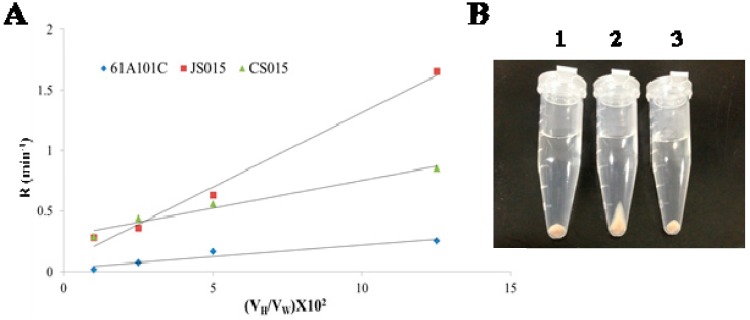
Cell surface hydrophobicity (**A**) and cell pellet pattern (**B**) of three *B. japonicum* strains: 1, 61A101C; 2, JS015; 3, CS015. (**A**) The *x*-axis represents removal rate per min as a function of the hexadecane-to-water volume ratio (*V*_H_/*V*_W_), while the *y*-axis represents hexadecane-to-water volume ratios (*V*_H_/*V*_W_).

Our previous studies on the LPS-deficient mutants revealed that their cell surface was more hydrophobic than that of the wild type [2,18]. As expected, JS015 also had increased hydrophobicity of its cell surface compared to the wild type and its complemented strain CS015 (Figure 3A). This result suggests that *O*-antigen composition of LPS is important to maintain a physiological nature of the *B. japonicum* cell surface, which is crucial in the intimate interaction between the bacterium and its host soybean (see, Section 2.6 nodulation result). Interestingly, when we observed cell pellet patterns after centrifugation, the patterns between JS015 and wild-type cell pellets were quite discernable. JS015 had a relatively long cell pellet pattern compared to the wild type and CS015 (Figure 3B). This result is consistent with cell pellet patterns of the previous LPS-deficient mutants [2,18].

### 2.4. The waaL Mutant Is Less Motile

**Figure 4:**
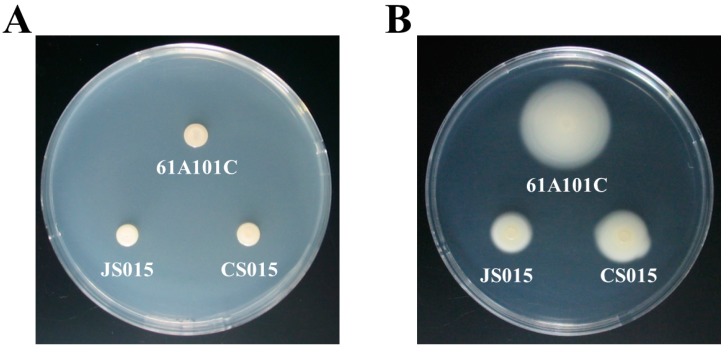
Motility of three *B. japonicum* strains 61A101C, JS015, and CS015: (**A**) 1% solid agar medium; and (**B**) 0.3% soft agar medium.

**Figure 5:**
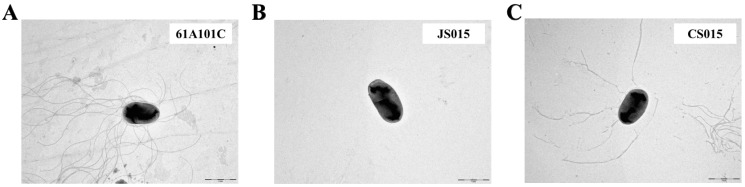
Electron microscopic analysis of flagella from the wild type (**A**); the *waaL* knock-out mutant (**B**); and its complemented strain (**C**). Bars = 5 µm.

In addition to surface hydrophobicity, other surface properties such as attachment and motility can be affected by the lack of *O*-antigen [27]. Thus, we compared motility among the wild type, JS015, and CS015 using the soft agar (0.3%) assay. JS015 showed significantly less motility than the wild type and CS015, even though the phenotype of CS015 was not fully restored (Table 1 and Figure 4B). We also carried out the control experiment using 1% solid agar plates, since the different growth rates between the wild type and JS015 may affect the motility. Generation times of the wild type, JS015, and CS015 were 8.5 ± 0.3, 11.8 ± 0.4, and 9.3 ± 0.8 h, respectively. Regardless of the growth rate, there was no difference among the three strains in the control experiment (Figure 4A). These results confirm that the decreased swimming motility in JS015 was due to defect in motility function rather than the slower growth rate. However, we wondered whether it was due to the loss of flagella or physical/structural regulation in motility apparatus (e.g., flagellum is intact, but its motion was limited by incomplete LPS and/or increased hydrophobicity). To determine this, the morphology of the three strains was observed by transmission electron microscopy (TEM) (Figure 5). The wild type shows several polar flagella, while JS015 appears to have no flagellum in its cell surface structure (Figure 5). Interestingly, CS015 restored some flagella, but not all, which explains partial complementation in the swimming motility assay. These results indicate that the decreased swimming motility by the disruption of the *waaL* gene in *B. japonicum* was due to a defect in flagella production. Our finding is also consistent with an observation made by Abeyrathne *et al.* [23], in that in *P. aeruginosa*, the lack of WaaL resulted in reductions in swimming and twitching motility and flagella production. Several other studies have also shown impaired motility in *waaL* mutants of *V. fischeri* [22], *E. amylovora* [24], and *Proteus mirabilis* [26].

**Table 1:** Characteristics of three *B. japonicum* strains in motility, nodulation, and stress responses.

Strains	Motility (mm in Diameter)	Osmotic Stress ^a^ (% Survivability)	Oxidative Stress ^b^ (Zone Inhibition Size in mm)	Novobiocin ^a^ (% Survivability)	Nodulation ^c^
61A101C	33.0 ± 1.5 A	106.4 ± 5.1 A	30.7 ± 0.4 A	92.5 ± 3.4 A	+
JS015	12.3 ± 0.9 B	94.2 ± 4.2 B	39.0 ± 0.7 B	64.7 ± 9.0 B	−
CS015	20.8 ± 0.6 C	109.2 ± 7.1 A	34.7 ± 2.2 A	88.7 ± 7.1 A	+

Values followed by different capital letters are statistically significant (*p* < 0.05) based on Student’s *t* test. Three biological replicates were used for each experiment except for nodulation assay. ^a^ Osmotic (50 mM NaCl) and novobiocin (20 µg·mL^−1^) stresses were calculated based on the initial cell number (100%); ^b^ Oxidative stress was calculated based on zone inhibition size after application of 5 µL of 3% (*v*/*v*) H_2_O_2_ onto the filter disk; ^c^ Nodule numbers were counted after 4 weeks of soybean growth. + indicates successful nodule formation, while − indicates there is no nodule formation. Plants inoculated with 61A101C or CS015 formed *ca.* 8 nodules per plant, whereas no nodules were formed in plants inoculated with JS015.

### 2.5. The waaL Mutant Is More Sensitive to Oxidative, Osmotic, and Novobiocin Stresses

Rhizobia may be exposed to various types of stresses before and during the nodulation process, including nutrient limitation, oxidative stress, and changes in osmolarity as well as toxic molecules [28,29,30,31,32]. For successful nodule development, it is essential that bacteria should survive and/or maintain in critical numbers for a long time in the rhizosphere or within the host plant [33]. Despite the mutual interaction in the symbiosis, host plants produce an oxidative burst (e.g., hydrogen peroxide) as a defense mechanism [34]. Thus, rhizobia have to be able to resist the plant’s innate defense mechanism to initiate nodulation. In addition to oxidative stress, salinity is another environmental factor that leads to reduced nodulation process [35].

Osmotic stress also affects root hair deformation and induces cell death [36]. To investigate the effect of the *waaL* gene on bacterial stress response to oxidative and osmotic stresses, we compared survivability of CS015 with that of JS015 when they were exposed to those stresses. JS015 showed more susceptibility to both oxidative and osmotic stresses as compared to the wild type and CS015 (Table 1). These results indicate that the lack of *O*-antigen in LPS affects bacterial sensitivity to hydrogen peroxide and survivability to osmotic stress.

In most Gram-negative bacteria, LPS can provide an effective permeability barrier against small hydrophobic molecules (e.g., hydrophobic antibiotics), allowing bacterial growth and survival in an unfavorable environment. We evaluated whether mutation on the *waaL* gene could affect bacterial susceptibility to novobiocin, a hydrophobic antibiotic that was used to isolate novobiocin-supersensitive (NS) mutants of *E. coli* [37]. In *E. coli*, most of NS mutants were found to have defective LPS, specifically such as a lack of external outer-core oligosaccharides. Similarly, high sensitivity to novobiocin was observed in JS015 compared with the wild type and CS015 (Table 1). These results indicate not only that alteration in LPS composition caused by mutations leads to the increased sensitivity to novobiocin, but also that *O*-antigen could be the primary structural constituent responsible for the function of LPS as a permeability barrier.

### 2.6. waaL Is Required for Successful Nodule Formation on Soybean Roots

Since a number of studies showed failure in nodulation of rhizobial LPS mutants deficient in *O*-antigen [10,12], we tested whether JS015 also failed to form mature nodules on soybean roots. Unlike the wild type, JS015 was not able to develop nodules (Figure S1). However, CS015 restored the ability to form functional nodules (Table 1). This result indicates that WaaL is one of the key players to induce and maintain a symbiotic relationship in nitrogen fixation between soybean and its symbiont *B. japonicum*.

## 3. Experimental Section

### 3.1. Bacterial Strains, Plasmids, and Growth Conditions

The bacterial strains and plasmids used in this study are described in Table 2. The *B. japonicum* strains were aerobically cultured in an AG medium [38] at 30 °C. The *E.* *coli* strains were aerobically cultured in Luria–Bertani (LB) medium at 37 °C. Antibiotics were used at the following concentrations when needed: chloramphenicol, 30 µg·mL^−1^; kanamycin, 150 µg·mL^−1^; streptomycin 100 µg·mL^−1^ for *B. japonicum* and ampicillin, 50 µg·mL^−1^; kanamycin, 50 µg·mL^−1^; and streptomycin, 50 µg·mL^−1^ for *E. coli*.

**Table 2:** Bacterial strains and plasmids.

Strains/Plasmids	Relevant Characteristics	Reference or Source
*B. japonicum*		
61A101C	Wild type, Cm^r^	Nitragin (Milwaukee, WI, USA)
JS015	Strain 61A101C *waaL*::Km, Km^r^	This study
CS015	JS015/pBBRL, Sm^r^, Km^r^	This study
*E. coli*		
DH5α	*supE44*, *ΔlacU169* (*Φ80 lacZΔM15*), *hsdR17*, *recA1*, *endA1*, *gyrA96*, *thi-1*, *relA1*	Invitrogen (Carlsbad, CA, USA)
S17-1	*recA pro* (RP4-2Tet::Mu Kan::Tn7)	[39]
**Plasmids**		
pGEM-T easy	PCR cloning vector, Amp^r^	Promega (Madison, WI, USA)
p34s-Km	Vector containing Km^r^ cassette, Km^r^	[40]
pTRL	pGEM T easy vector containing 1.5 kb fragment of *waaL* gene, Amp^r^	-
pTRLK	pTRL derivative *waaL* gene disruption by deletion of BssHII fragment and insertion of Km^r^ cassette, Km^r^	-
pJQ200SK	Suicide vector, *sacB*, Gm^r^	[41]
pJQRLK	pJQ200SK-*waaL*::Km, Gm^r^, Km^r^	This study
pIJ778	pBluescript KS(+) derivative containing the streptomycin/spectinomycin resistance gene *aadA*, Sm^r^	[42]
pBBR1MCS-2	Broad host range expression vector, *mod*, *rep*, *lacZ*, Km^r^	[43]
pBBRL	pBBR1MCS-2 carrying the 1.5 kb fragment of *waaL* gene, Km^r^	This study
pBBRL-Sm	pBBRL derivative containing the Sm^r^ gene, Km^r^ gene disruption by deletion of NcoI fragment, Sm^r^	This study

### 3.2. DNA Sequence Analysis

Restriction enzymes were purchased from TaKaRa Biotechnology (Dalian, China) and were used according to the supplier’s specifications. Plasmid DNA was extracted with a LaboPass™ plasmid DNA purification kit (Cosmo Genetech, Seoul, Korea). When needed, DNA was purified from the agarose gel by using a LaboPass™ Gel Extraction Kit (Cosmo Genetech, Seoul, Korea). The T4 DNA ligase was purchased from TaKaRa Biotechnology (Dalian, China). DNA was quantified using a Nanodrop Technologies ND-1000 spectrophotometer (Nano-Drop Technologies, Wilmington, DE, USA). Sequence homology searches were performed by using the BLAST algorithm at the National Center for Biotechnology Information. Hydropathy profiles of various WaaL proteins were plotted using the Kyte–Doolittle algorithm with a window size of 9 [44]. The nucleotide sequence of the *B. japonicum waaL* gene has been submitted to GenBank under the accession number KC172899.

### 3.3. Construction of a waaL Mutant and Its Complemented Strain

A *waaL* mutant strain was constructed by deletion of the gene and insertion of a kanamycin-resistance cassette into the coding sequence using the *sacB*-based suicide vector pJQ200SK [41]. Briefly, a 1.5-kb fragment containing the *waaL* gene and its upstream and downstream regions was PCR-amplified and subcloned into the pGEM-T Easy vector (Promega, Madison, WI, USA), resulting in pTRL. Then, the *waaL* gene was disrupted by deleting the internal 220-bp BssHII fragment spanning 55th and 274th nucleic acid positions and replacing it with the 1.2-kb kanamycin-resistance cassette from p34S-Km [40], which results in pTRLK. The disrupted *waaL* gene fragment was then cloned into the PstI site in pJQ200SK, creating pJQRLK. With bi-parental mating, pJQRLK was transferred from *E. coli* S17-1 to *B. japonicum* 61A101C. Lastly, the *waaL* mutant strain created by double-crossover recombination was selected on AG agar plates containing chloramphenicol (30 µg·mL^−1^), kanamycin (150 µg·mL^−1^), and 5% sucrose. The *sacB* gene of pJQ200SK was used as a positive selection marker for double-crossover recombination events against single-crossover recombination. The resulting *waaL* mutant was named JS015 and its gene disruption was confirmed by colony PCR.

To construct a *waaL*-complemented strain, the *waaL* gene fragment excised from pTRL using KpnI and XbaI was cloned into KpnI-XbaI sites of pBBR1MCS-2 [43], creating pBBRL. Then, the 1.3-kb streptomycin-resistance cassette from pIJ778 [42] was inserted into the SacI site of pBBRL and part of the kanamycin cassette was removed by deletion of the 401-bp NcoI fragment, resulting in the replacement of kanamycin cassette with streptomycin-resistance gene. The resulting plasmid pBBRL-Sm was introduced into JS015 by bi-parental mating. The complemented strain was named CS015 and verified by colony PCR.

### 3.4. LPS Profile Analysis

LPSs were extracted as described by Carrion *et al.* [9] and analyzed by sodium dodecyl sulfate-polyacrylamide gel electrophoresis (SDS-PAGE). After SDS-PAGE, LPSs were visualized on a gel strained with Pro-Q^®^ Emerald 300 LPS stain kits purchased from Molecular Probes (Eugene, OR, USA).

### 3.5. Cell Surface Hydrophobicity Analysis

Hydrophobicity on the cell surface was determined by the microbial adhesion to hydrocarbon (MATH) assay as described previously [45]. Hexadecane was used as a hydrocarbon source and the percentage of MATH was calculated as follows: % MATH = {(*A*_0_ − *A*_t_)/*A*_0_} × 100, where *A*_0_ is the initial optical density and *A*_t_ is the optical density of the cell suspension at time *t*.

### 3.6. Motility and Transmission Electron Microscope (TEM) Analysis

*B. japonicum* cells were harvested at mid-log phase by centrifugation at 8000× *g* and washed with phosphate-buffered saline (PBS, pH 7.0). All strains of *B. japonicum* were then resuspended in PBS to adjust OD_600_ = 0.5. Aliquots of 5 µL of the bacterial suspensions were placed on the 0.3% AG agar plate and incubated for 7 days at 30 °C. The motility was quantified by measuring the diameter of cell diffusion. To observe the bacterial flagella, TEM images of the *B. japonicum* cells were obtained with a LEO 912 AB energy-filtered TEM (Carl Zeiss Inc., Berlin, Germany, Korea Basic Science Institute, Chuncheon, Korea) operated at 120 kV. Cells were placed on the grids and stained for 10 min each with 4% uranyl acetate, washed with distilled water, air-dried, and examined under the TEM.

### 3.7. H_2_O_2_ Inhibition Zone Assay

A filter disk assay was performed as described by Jeon *et al.* [31] with modification. *B. japonicum* cells exponentially grown in AG broth were collected by centrifugation (6000× *g*, 10 min, 4 °C), washed twice with PBS, and adjusted to an OD_600_ of 1.0. Then, 100 µL of the cell suspension was spread on AG agar plates. A sterilized filter disk of 5 mm in diameter was placed in the center of the agar plates, and 5 µL of 3% (*v*/*v*) H_2_O_2_ was applied onto the filter disk. The inhibition zones were measured in millimeters after the plates were incubated at 30 °C for 7 days.

### 3.8. Osmotic Stress and Novobiocin Sensitivity Assay

Sensitivity of *B. japonicum* cells to osmotic stress and novobiocin was evaluated by viable cell count (e.g., CFU·mL^−1^). Cell suspension with an OD_600_ of 1.0 prepared as described above was 10-fold serially diluted (10^−1^ to 10^−6^). Then, 10 µL of each diluted aliquot was spotted onto AG agar plates containing 50 mM NaCl or 20 µg·mL^−1^ novobiocin. After 7 days of incubation at 30 °C, CFUs were counted and % survivability was calculated based on the initial cell number as 100%.

### 3.9. Nodulation Assay

Laboratory-scale nodulation assays were performed using plastic growth pouches (Mega International, Minneapolis, MN, USA). As described by Lee *et al.* [46], soybean seeds (*Glycine max* L. cv. Williams) were surface-sterilized and germinated in petri dishes for 2–3 days. Each seedling, with a root length between 2 and 3 cm, was aseptically placed in autoclaved pouches and inoculated with 1 mL of the bacterial suspension (OD_600_ = 0.1) of 61A101C, JS015, or CS015. The seedlings were grown in a growth chamber with 16 h light/8 h dark at 26 °C and watered with half-strength Broughton & Dilworth (B&D) medium [47] when needed. A total of 18 plants were harvested after 4 weeks of growth, and nodule numbers were counted.

## 4. Conclusions

In this study, JS015 exhibited altered phenotypes including incomplete LPS, increased surface hydrophobicity, decreased motility, and increased sensitivity toward hydrogen peroxide, osmotic stress, and novobiocin. Among them, incomplete LPS resulting from inactivation of the *waaL* gene likely affected the outcome of the other phenotype changes. Ultimately, lack of *O*-antigen in the *B. japonicum* LPS has an immediate and vital influence on failure of nodulation in soybeans. Our findings support the notion that (i) *O*-antigen ligase encoded by *waaL* is a key enzyme in the *B. japaponicum* cell wall biosynthesis, which links the *O*-antigen to the outer core of LPS and (ii) intact rhizobial LPS is important not only to establish successful symbiotic relationships, but also to provide resistance against a host defense mechanism, such as oxidative burst, and other environmental fluctuations.

## Supplementary Materials

Supplementary materials can be found at http://www.mdpi.com/1422-0067/16/08/16778/s1.
