# Supplementary material for: Effects of the Bradyrhizobium japonicum waaL (rfaL) Gene on Hydrophobicity, Motility, Stress Tolerance, and Symbiotic Relationship with Soybeans
Source: Int J Mol Sci. 2015 Jul 23;16(8):16778–91. doi: 10.3390/ijms160816778 (PMC4581169; doi:10.3390/ijms160816778)
Supplement: Supplementary file 1 [file ijms-16-16778-s001.pdf]

Supplementary Information

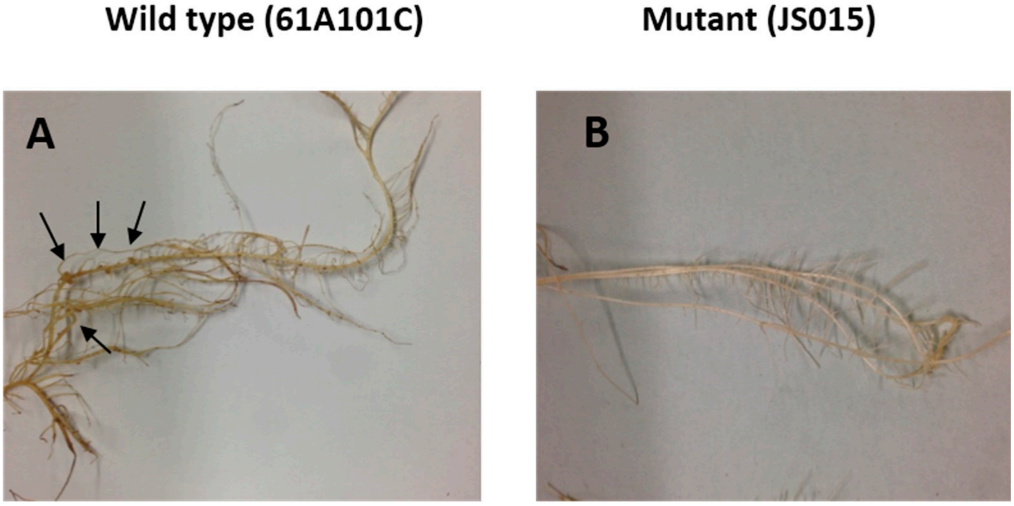

Figure S1. Representative images of soybean roots inoculated with either the wile type (**A**) or JS015 (**B**). Plants were harvested after 4 weeks of growth. Arrows indicate nodules formed on soybean roots. Note that there is no nodule formed on roots with JS015 as shown in (**B**).
